# Hyaluronic acid functionalized ZnO nanoparticles co-deliver AS and GOD for synergistic cancer starvation and oxidative damage

**DOI:** 10.1038/s41598-022-08627-w

**Published:** 2022-03-17

**Authors:** Zhenkun Ren, Xibin Han, Lixin Wang, Yi Wang

**Affiliations:** 1 The Third Affiliated Hospital of Jinzhou Medical University, Jin Zhou, 121000 People’s Republic of China; 2 The Laboratory Animal Center, Jinzhou Medical University, Jin Zhou, 121000 People’s Republic of China; 3 Husbandry and Veterinary Academy, Jinzhou Medical University, Jin Zhou, 121000 People’s Republic of China

**Keywords:** Cancer, Cell biology

## Abstract

Artesunate was reported to have inhibition effect on tumors via amplified oxidative stress while the lack of intratumoral ferrous ions supply greatly hinders its efficacy. Herein, the AS/GOD@HAZnO NPs we proposed could be efficiently taken in by the affinity between hyaluronic acid and the CD44 receptors. DLS and TEM results manifested the nano-size (~ 160 nm) and circular shape of AS/GOD@HAZnO NPs. Due to the acid-responsive degradation, AS/GOD@HAZnO NPs realized responsive release (up to 80%) in acid environment while only 20% was released in neutral medium. The cellular and in vivo experiment showed that co-delivery of AS and GOD via HAZnO NPs could effectively induce the overproduction of ROS and cut the glucose supply of tumor cells, and thus result in efficient cell apoptosis and tumor inhibition.

## Introduction

In 2015, the Nobel Prize in Physiology or Medicine was awarded to Youyou Tu for her discovery and anti-malaria research of artemisinin. From then on, artemisinin and its derivatives are increasingly focused and explored. In addition to the effective treatment of malaria, artemisinin and its derivatives also exhibited a robust inhibition effect on cancer cells. As a typical derivative of artemisinin, artesunate (AS) was proved surprisingly conducive to suppress tumor growth and widely investigated for novel anti-cancer strategies^1^. It is universally accepted that the intrinsic peroxide bridge of AS can be broken by the endogenous ferrous ions in tumor cells via a Fenton-like reaction and release reactive oxygen species (ROS), which then leads to overwhelming oxidative stress^2^. Meanwhile, the excessive oxidative substances easily cause damages to the proteins, lipids, and nucleic acids and thus induce apoptosis of tumor cells^3,4^. Of note, AS owns a large natural source and tremendous safety profiles from anti-malaria practices that make it quite promising in anti-cancer applications.

Whereas, due to the insufficient supply of ferrous ions within tumor cells, AS cannot effectively experience the breakage of peroxide bridge or produce too much ROS^5^. This deficiency significantly reduced the anti-cancer efficacy and raised an obstacle for scholars. Mainstream research focused on supplying AS with exogenous ferrous ions via Fe-based nanocarriers while the intratumor pH is not optimal to excavate the best performance of Fenton-like reaction^6^. On the other hand, monotherapy usually fails to overcome tumor heterogeneity and the multiple pathways involved in tumor pathogenesis^7^. Overall, it is highly necessary to establish novel multimodal therapies to enhance the anti-cancer effect of AS.

Among various nano-functional materials, zinc oxide nanoparticles (ZnO NPs) have been widely used in the food, cosmetic and medical industries^8^. Recently, ZnO NPs have been attracting vast interest for their intriguing ability to induce oxidative stress, inflammation effect, and even cell death to cancer cells^9,10^. It is reported that ZnO NPs could high-efficiently enter lysosomes via the autophagy pathway and rapidly release zinc ions due to the acidic environment. Subsequently, zinc ions would damage mitochondria and lysosomes, further disrupting the negative feedback mechanisms between ROS and mitophagy, leading to damaged mitochondria accumulation, excessive ROS production and cell death^11,12^. Other reports also proved that ZnO NPs could produce ROS and oxidative damage by sequential oxidation–reduction reactions occurring at particles’ surface. Therefore, cancer cells could be led to severe death for that ROS are common mediators for cell apoptosis^13–15^. To sum up, ZnO NPs are ideal nanocarriers to AS for they can realize the synergistic enhanced production of ROS and amplification of oxidative damage.

Considering that tumor’s strong ability of metabolism compensation may counter the redox imbalance brought by excessive ROS, it is better to combine other therapies which can interrupt the intratumoral metabolism, to ensure the effective inhibition on tumor cells. According to the Warburg effect, tumor cells are more dependent on glucose nutrients than normal cells; unlike their normal counterparts, tumor cells utilize glycolysis instead of mitochondrial oxidative phosphorylation for glucose metabolism even in oxygen-rich conditions^16^. Therefore, starvation therapy that uses glucose oxidase (GOD) to decompose glucose into H_2_O_2_ and gluconic acid and block tumor’s nutrients supply is considered as a promising treatment strategy^17,18^. The generated H_2_O_2_ can enter tumor organelles via aquaporins and cause oxidative damages, yielding an apparent antitumor effect^19,20^. Meanwhile, continuous accumulation of H_2_O_2_ and the contribution to acidic environment (gluconate) tend to accelerate AS’s Fenton-like reactions, thereby increasing oxidative damage^21^. Besides, the membrane-associated catalase of tumor cells can promote the catalysis of H_2_O_2_ to produce O_2_, which in turn accelerates GOD catalysis and enhances cancer starvation therapy^22,23^.

**Figure 1:**
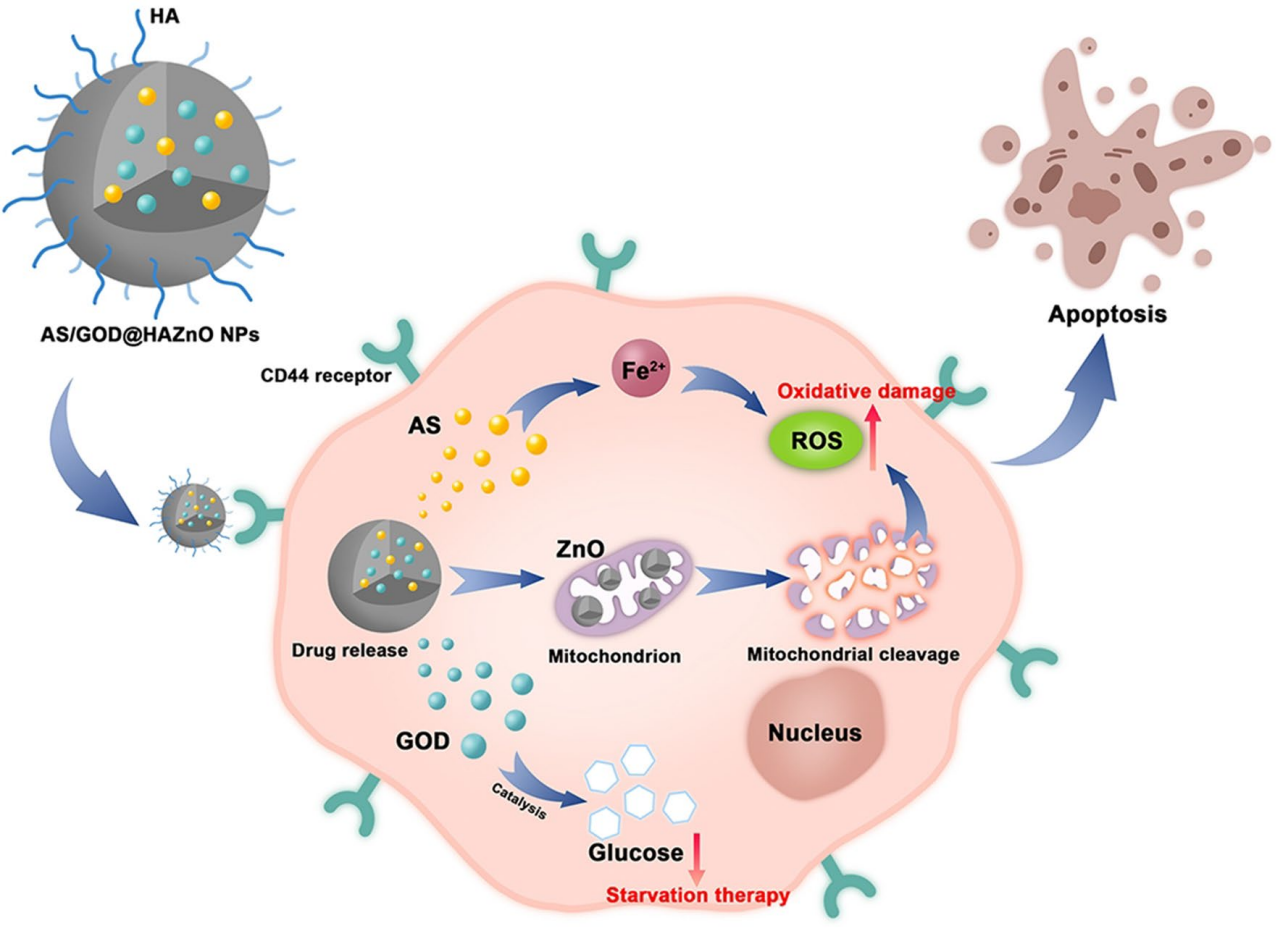
The schematic illustration of AS/GOD@HAZnO NPs’ entering tumor cells and releasing drugs. The oxidative damage and starvation therapy were described using the oxidation of AS, mitochondrial cleavage, and glucose catalysis.

Herein, we designed hyaluronic acid (HA)-functionalized ZnO NPs to co-deliver AS and GOD. The HA layer can increase the active targeting ability of ZnO NPs through the intrinsic affinity with CD44 receptors on tumor cells. As shown in Fig. 1, ZnO NPs can be partially decomposed by the acidic environment and release the loaded AS and GOD after entering the tumor cells. ZnO NPs and AS would induce the overproduction of ROS and lead to oxidative damage on tumor cells. Meanwhile, GOD can catalyze glucose into gluconic acid and H_2_O_2_, blocking tumor’s nutrient supply and producing cancer starvation. What’s more, the generated H_2_O_2_ can be catalyzed into O_2_ which in turn enhances GOD catalysis and starvation therapy.

## Results and discussion

### Characterization of AS/GOD@HAZnO NPs

**Figure 2:**
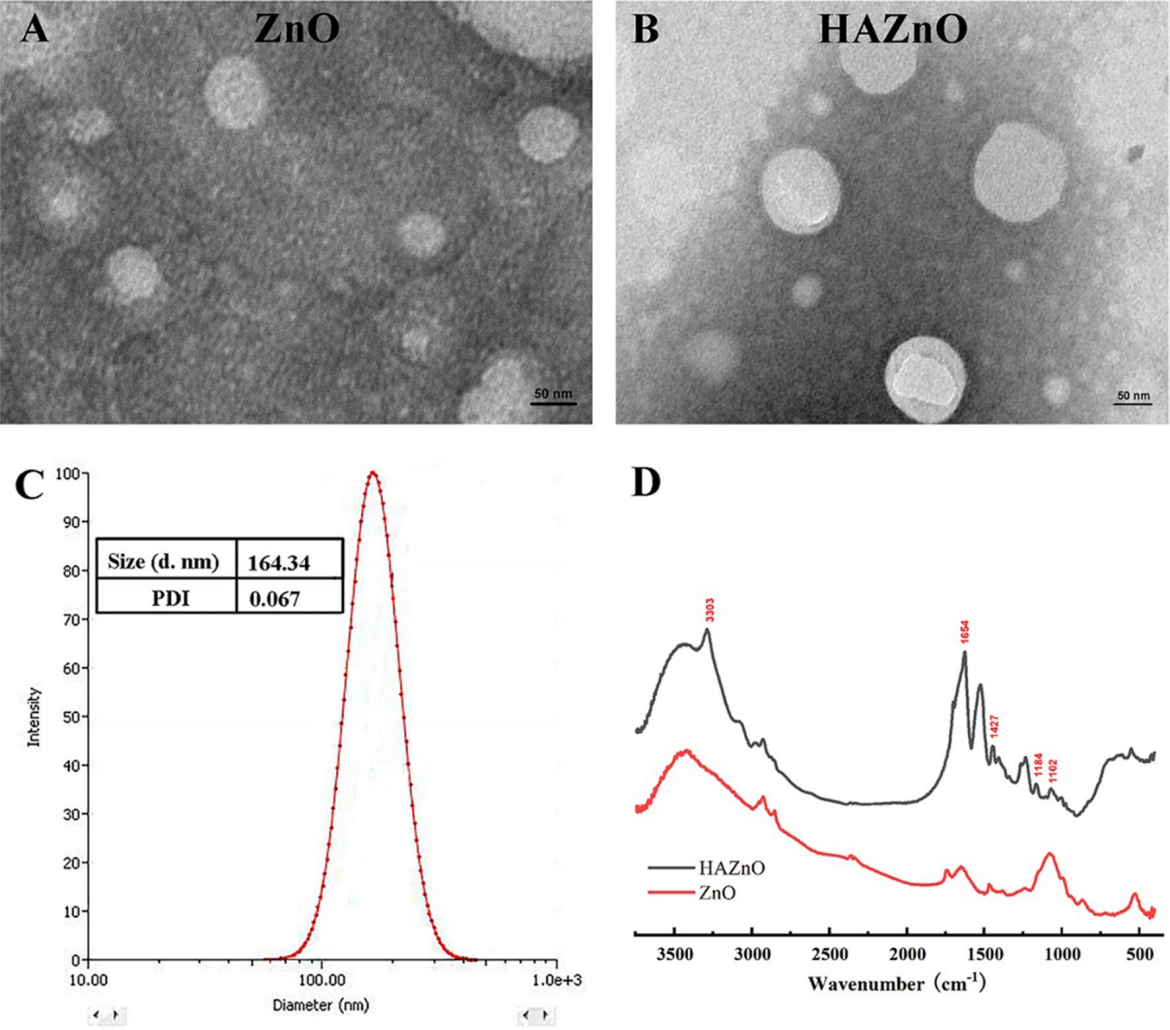
(**A**) TEM image of ZnO NPs; (**B**) TEM image of HAZnO NPs; (**C**) Size distribution of HAZnO NPs (DLS measurement); (**D**) FTIR spectrum of ZnO and HAZnO NPs.

The interaction between HA and AS/GOD@ZnO could be explained by the intermolecular hydrogen bonding force or van der Waals force between them^24^. According to supporting information Table S1, the weight ratio of HA to AS/GOD@ZnO applied for the preparation of AS/GOD@HAZnO in this study was chosen to be 4:1. The size of the optimized AS/GOD@HAZnO nanoparticles was found to be 163.33 ± 5.65 nm with a PDI of 0.10 ± 0.04. The size, zeta potential, and PDI value of HAZnO and ZnO were shown in the Table S2. As shown in Fig. 2A,B, the prepared ZnO NPs has proper uniformity and displayed a size of ~ 150 nm. After the functionalization of HA, NPs did not show any significant changes and maintained circular shape, while the typical peaks of glycosyl in the HA structure (1102 cm^−1^ and 1184 cm^−1^) from FTIR spectra (Fig. 2D) indicates the existence of HA. Besides, peaks at 1427 cm^−1^ and 1654 cm^−1^ belong to the symmetrical and asymmetrical stretching peaks of –COOH, which should be ascribed to the carboxyl group in HA structure. In addition, DLS result (Fig. 2C) confirmed the nano-size (164.34 nm) and good uniformity (PDI = 0.067) of HAZnO NPs. After the degradation of the main structure of AS/GOD@HAZnO NPs, the loading capacity of AS and GOD were measured and calculated as 5.77 ± 1.31% and 2.29 ± 0.31%, respectively. Moreover, according to DLS analysis, the nanocarriers HAZnO were stable in PBS (pH 7.4) and PBS containing 10% FBS for three days at 37 °C (Fig. S1).

### AS release kinetics

**Figure 3:**
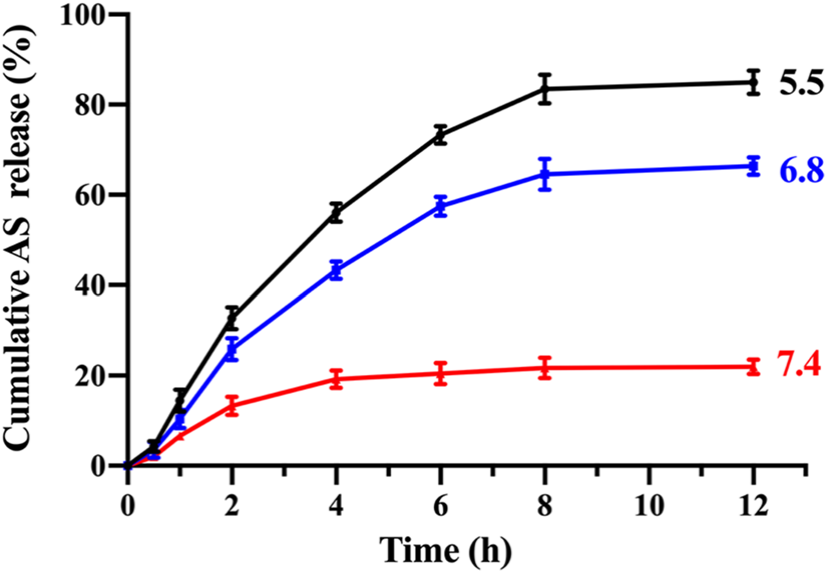
Cumulative AS release percentage against time in PBS with three different pH (5.5, 6.8, and 7.4). Data are presented as mean ± SD, n = 3.

Considering the ZnO NPs can decompose into water and zinc ions in acid conditions, the AS/GOD@HAZnO NPs are expected to have the acid-responsive drug release behavior. In pH 7.4 PBS (Fig. 3), AS slowly leaked out from the NPs and only reached 20% maximum release. In comparison, the increase in the proton concentration distinctly brought the release kinetics to a faster level and realized 60% and 80% maximum release in pH 6.8 and 5.5 conditions within 12 h. This characteristic corresponds to the acid-responsive decomposition of ZnO NPs and bring the intratumoral specific release to a world of possibilities.

### Catalytic activity

**Figure 4:**
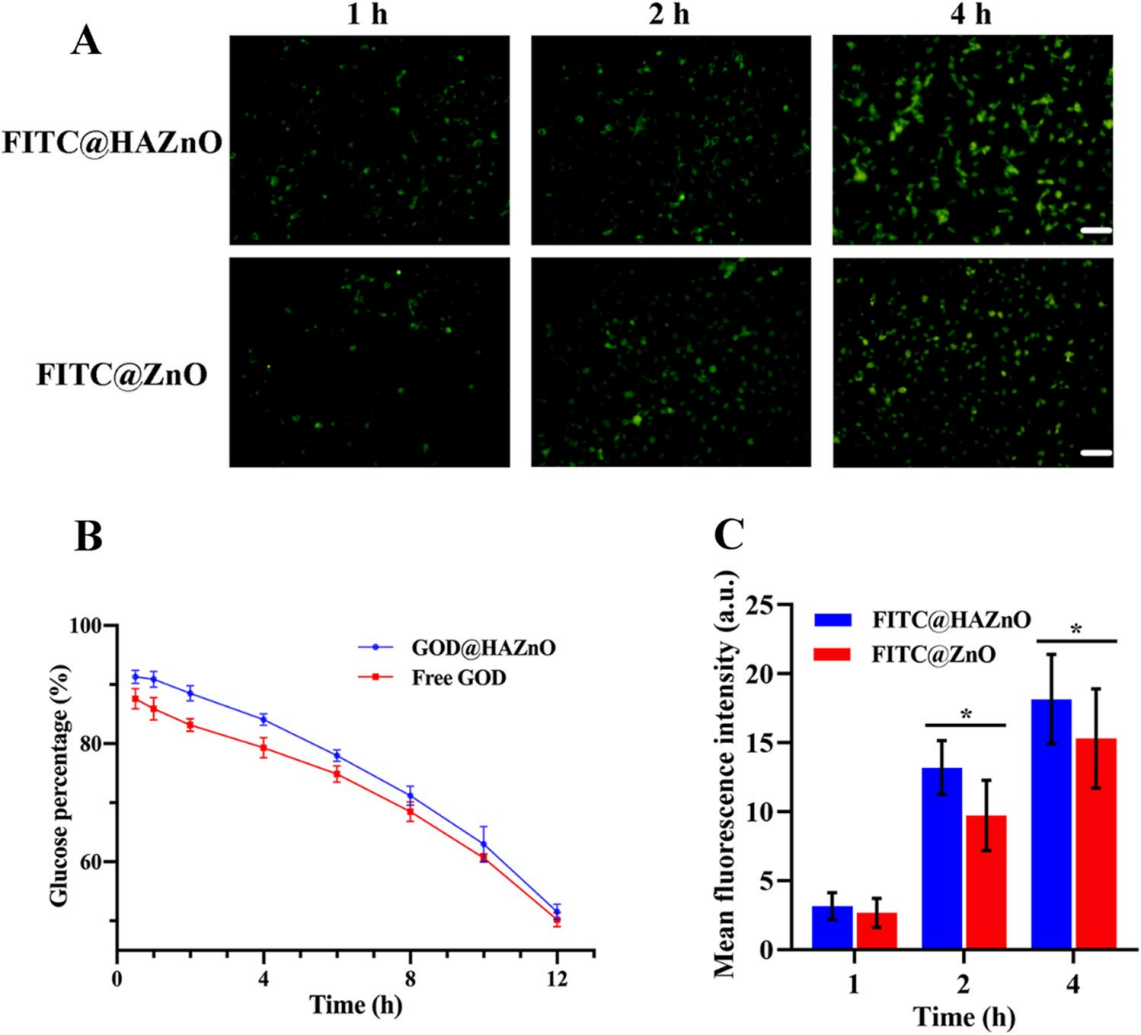
(**A**) Fluorescence images of 4T1 cells represent the intracellular uptake at 1, 2, and 4 h, respectively; (**B**) The remaining glucose percentage after co-incubated with GOD@ZnO or free GOD; (**C**) The semiquantitative analysis: mean fluorescence intensity of images in (**A**). Data are presented as mean ± SD, n = 3.

Of note, when AS/GOD@HAZnO NPs encounter glucose, they would initiate the degradation of glucose and block the nutrient supply of tumors, which is so-called starvation therapy. Therefore, the catalytic efficiency of AS/GOD@HAZnO NPs is the foundation of therapy. Our work not only assessed the efficiency against time but also compared AS/GOD@HAZnO NPs with free GOD to check whether the encapsulation would influence the activity of GOD. Figure 4B illustrated that within the first 6 h, free GOD exhibited faster catalytic kinetics than AS/GOD@HAZnO NPs probably because some GOD is lodged deeply in the HAZnO NPs and could not get exposed to glucose at the initial stage. Fortunately, two groups gave almost the same maximum glucose degradation (only 50% glucose left) at 12 h. In summary, although loading GOD in HAZnO NPs slowed the initial catalytic velocity, it did not decrease the catalytic capacity of GOD.

### Cellular uptake

It is reported that CD44 receptors are highly expressed in the 4T1 cells and HA can recognize CD44 with high affinity. Through the functionalization of HA, the cellular uptake of ZnO NPs is expected to be improved. The fluorescence images were shown in Fig. 4A and the semiquantitative bar chart was presented in Fig. 4C. Green fluorescence in FITC@HAZnO group was observed to be brighter than FITC@ZnO group within 4 h. The semiquantitative result also specified the difference and confirmed that there was obvious improvement in the cellular uptake of FITC@HAZnO NPs. This feature could help increase the efficacy of drugs delivered by HAZnO NPs and reduce the systemic toxicity of ZnO NPs.

To further confirm the CD44 receptor-mediated uptake and tumor-targeting ability of HAZnO, we selected 4T1 cells for receptor blockade experiments. The results showed that the green fluorescence of HAZnO in 4T1 cells was significantly reduced after HA pre-incubation (Fig. S2), which was due to the blocking of the CD44 receptor on the cell membrane by HA binding, resulting in reduced uptake of HAZnO NPs^25^. The above results indicate that HA is a key factor in identifying tumor cells, and HAZnO has good targeting ability to CD44-overexpressing tumor cells.

### Intracellular ROS assay

**Figure 5:**
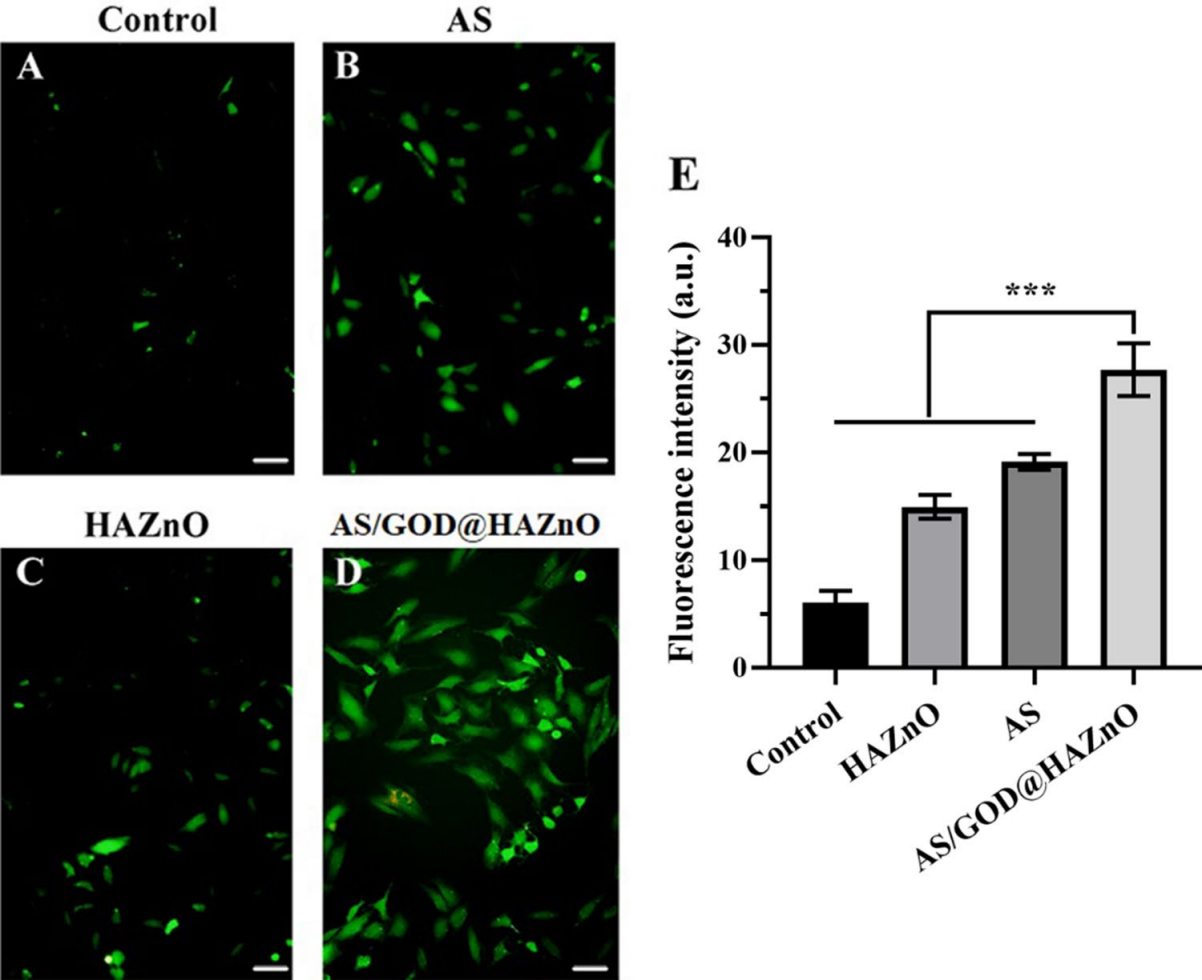
Fluorescence images indicate the intracellular ROS level of Control (**A**), AS (**B**), HAZnO (**C**), and AS/GOD@HAZnO (**D**) group; Scale bar = 100 μm. (**E**) The semiquantitative analysis of fluorescence images of ROS level. Data are presented as mean ± SD, n = 3.

**Figure 6:**
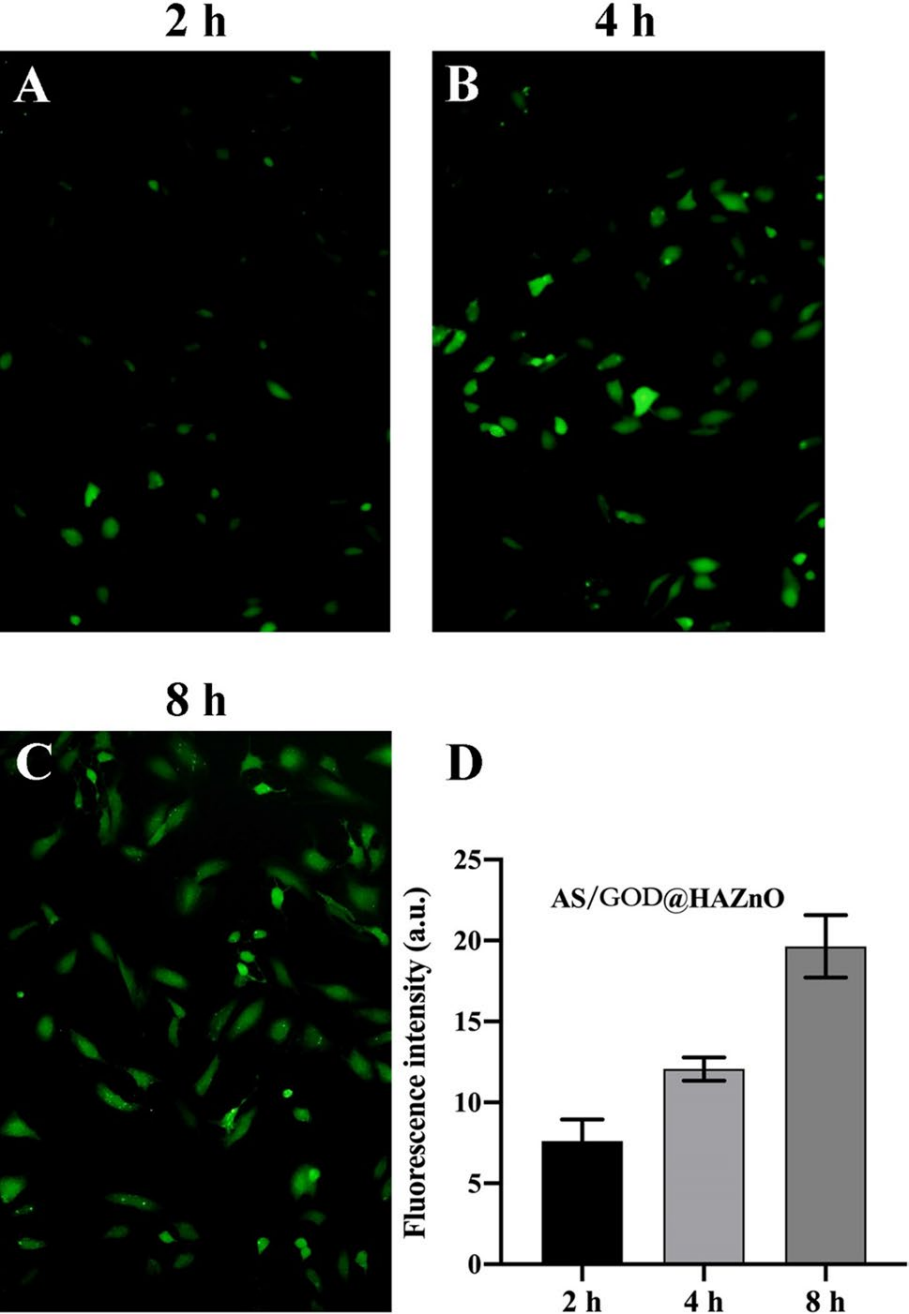
Fluorescence images indicate the intracellular ROS level of 4T1 cells after incubation with AS/GOD@HAZnO for 2 h, 4 h, and 8 h, respectively. Data are presented as mean ± SD, n = 3. Scale bar = 100 μm.

As discussed in the introduction, ROS plays a pivotal role in the cell death induced by AS and ZnO. The overproduction of ROS could destroy membrane lipids and mitochondrial DNA, thus leading to efficient cell apoptosis. During the co-incubation, AS and HAZnO both showed the ROS-inducing ability (Fig. 5) while AS is slightly stronger than HAZnO. Apparently, loading AS in HAZnO significantly increased the production of ROS, which supports that combining AS and ZnO can make up for the deficiency of intracellular ROS in an effective way and trigger robust amplification of oxidative stress. As shown in Fig. 6, the intensity of green fluorescence was increasing over time, suggesting the time-dependent and sustained behavior of ROS-inducing activity.

### In vitro cytotoxicity

**Figure 7:**
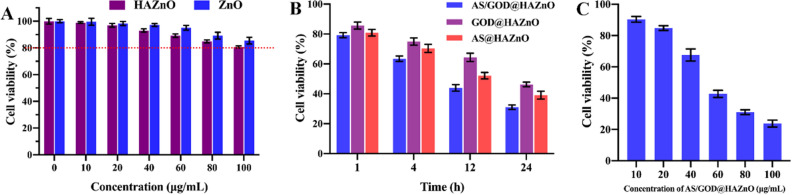
(**A**) Cell viability of 4T1 cells after treated with ZnO and HAZnO NPs for 24 h; (**B**) Cell viability of 4T1 cells after treated with AS/GOD@HAZnO, GOD@HAZnO, and AS@HAZnO NPs at different timepoints; (**C**) Cell viability of 4T1 cells after treated with different concentrations of AS/GOD@HAZnO for 24 h (10 ~ 100 μg/mL). Data are presented as mean ± SD, n = 3.

It is reported that the cytotoxicity of ZnO particles increases with decreasing the particle size and the nano-size ZnO might have obvious cytotoxicity^26^. It is confirmed that 4T1 cells lost 17% viability after being treated with 100 μg/mL ZnO NPs (Fig. 7A) in the MTT experiment. As the cellular uptake assessment revealed, the functionalization of HA improved the uptake of ZnO NPs. As a result, the HAZnO exerts more severe damage on 4T1 cells (Fig. 7A) than ZnO and induces 20% loss of cell viability. From Fig. 7B, it is suggested that co-delivering AS and GOD displayed higher efficiency of cytotoxicity through the synergistic function of ROS damage and starvation therapy. Besides that, the cytotoxicity of AS@HAZnO, GOD@HAZnO, and AS/GOD@HAZnO all abide by a time-dependent manner, indicating the long-lasting therapy efficacy of AS/GOD@HAZnO. Figure 7C demonstrated that at a relatively low dose (10 μg/mL), AS/GOD@HAZnO induced—cell damage was not obvious. The induced cell death gradually increased with increasing concentration, and when the concentration increased to 100 μg/mL, the cell viability decreased to only 23.76% ± 2.20%.

### Cell apoptosis

**Figure 8:**
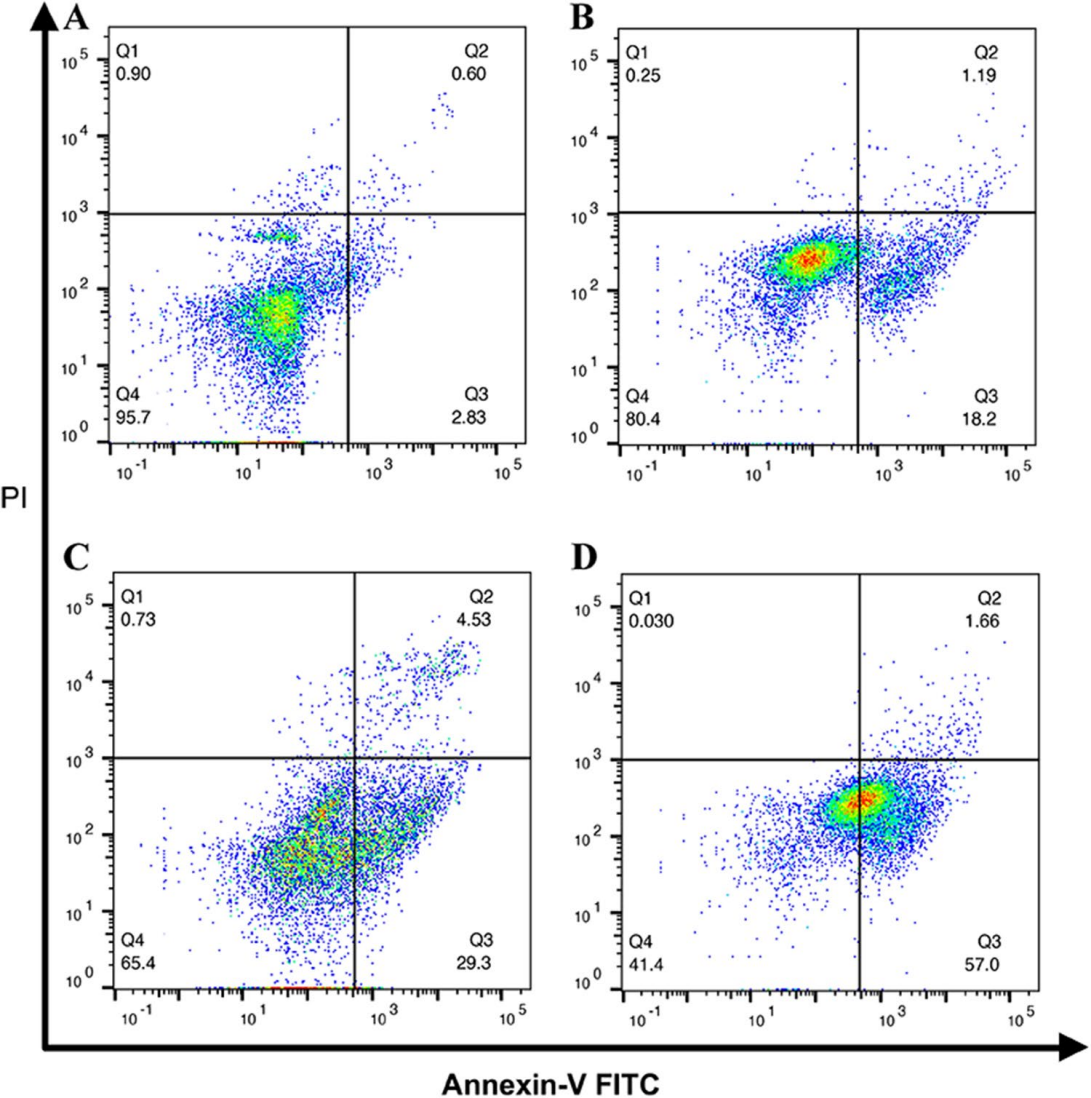
Flow cytometry analysis of cell apoptosis after treated with saline (**A**), GOD@HAZnO (**B**), AS@HAZnO (**C**), and AS/GOD@HAZnO NPs (**D**) for 24 h.

As the main cell death pathway of oxidative damage and starvation therapy, cell apoptosis becomes a vivid standard to assess the therapeutic efficacy of our AS/GOD@HAZnO NPs. Through the Annexin-V FITC/PI detection kit, the cell apoptosis was assessed, and the result was visualized in Fig. 8 and Table S3. Treated with GOD@HAZnO (Fig. 8B) and AS@HAZnO (Fig. 8C), 4T1 cells suffered distinct cell death and AS@HAZnO displayed stronger destruction effect (29.3% cell apoptosis) than GOD@HAZnO. Cells treated with AS/GOD@HAZnO suffered a remarkably higher apoptosis percentage (57.0%) and exhibited uniform apoptosis behavior compared to the other two groups. This indicates that the combination of starvation therapy and amplification of oxidative stress is reliable to enhance the chemotherapy of cancer.

### In vivo anti-tumor test

**Figure 9:**
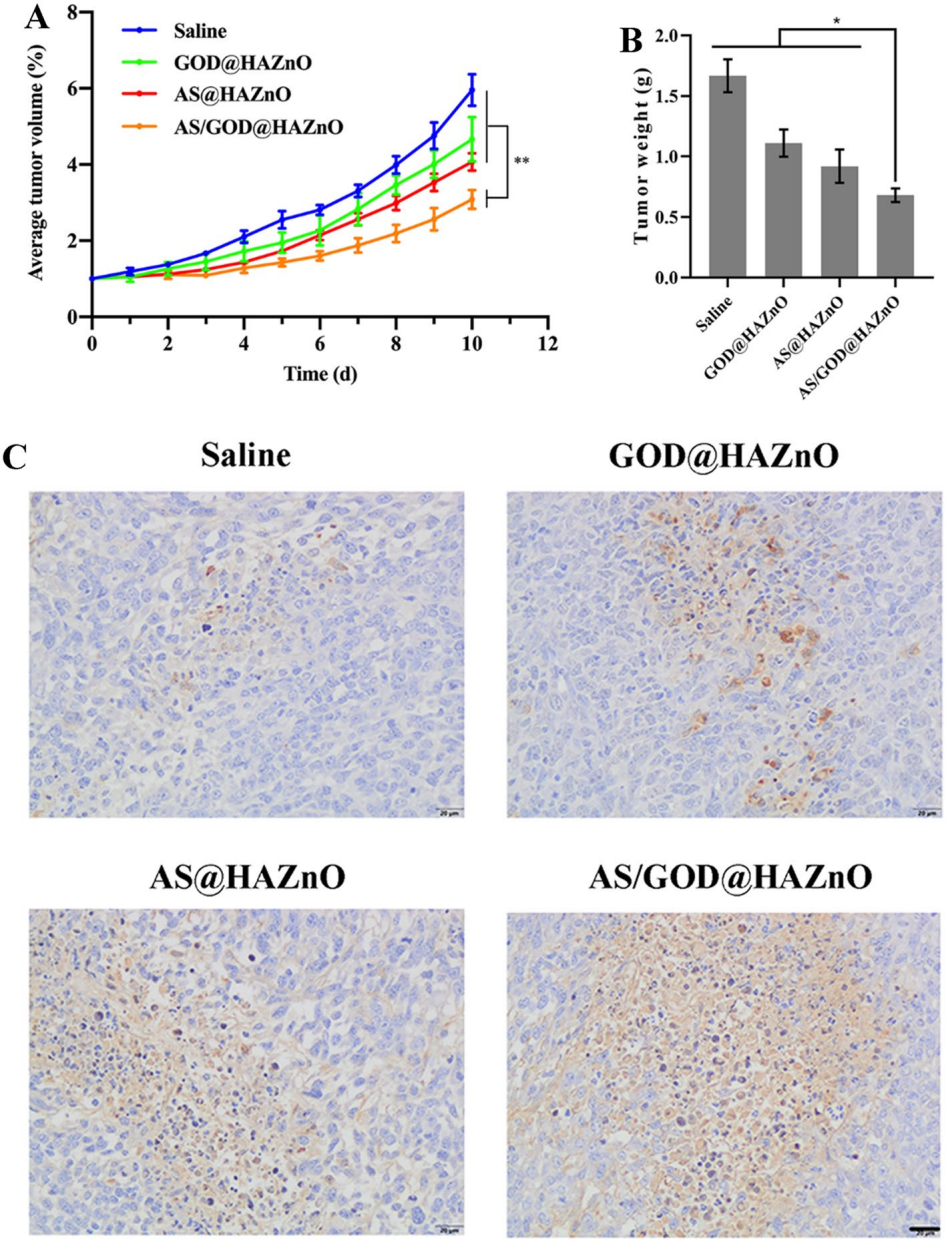
(**A**) Average tumor volume of tumor-bearing mice after treated with saline, GOD@HAZnO, AS@HAZnO, and AS/GOD@HAZnO; (**B**) The ultimate tumor weight of above groups; (**C**) The Tunel analysis of tumors from above groups. Scale bar = 20 μm. Data are presented as mean ± SD, n = 3. *P < 0.05, **P < 0.01, and ***P < 0.001.

The murine breast cancer models were established with 4T1 cells on BALB/c mice. During the administration, the tumor volume was keeping increasing, while AS@HAZnO and GOD@HAZnO group grows slower than the saline group, indicating that tumor growth was inhibited by AS@HAZnO and GOD@HAZnO (Fig. 9A). What’s more, tumors from AS/GOD@HAZnO group exhibited the slowest growth rate, significantly slower than the other groups. The ultimate tumor weight data (Fig. 9B) also demonstrated the same result that tumors from AS/GOD@HAZnO group has the smallest weight. These two results together showed that the HAZnO-based starvation therapy (GOD@HAZnO) and oxidative damage (AS@HAZnO) can perform a potent tumor inhibition effect while the combination of two therapies gave the significantly improved synergistic function.

In addition, the harvested tumors were also sent for Tunel analysis. DNA in dead cells or cells in apoptosis would break and expose their 3’-OH terminal which would be dyed by fluorescein and turn brown. Corresponding to the tumor weight data, GOD@HAZnO and AS@HAZnO induced apparent cell death (Fig. 9C). More cell death appeared in AS/GOD@HAZnO group, and cell junctions were severely destroyed, indicating that the tumor tissues have suffered potent damage.

**Figure 10:**
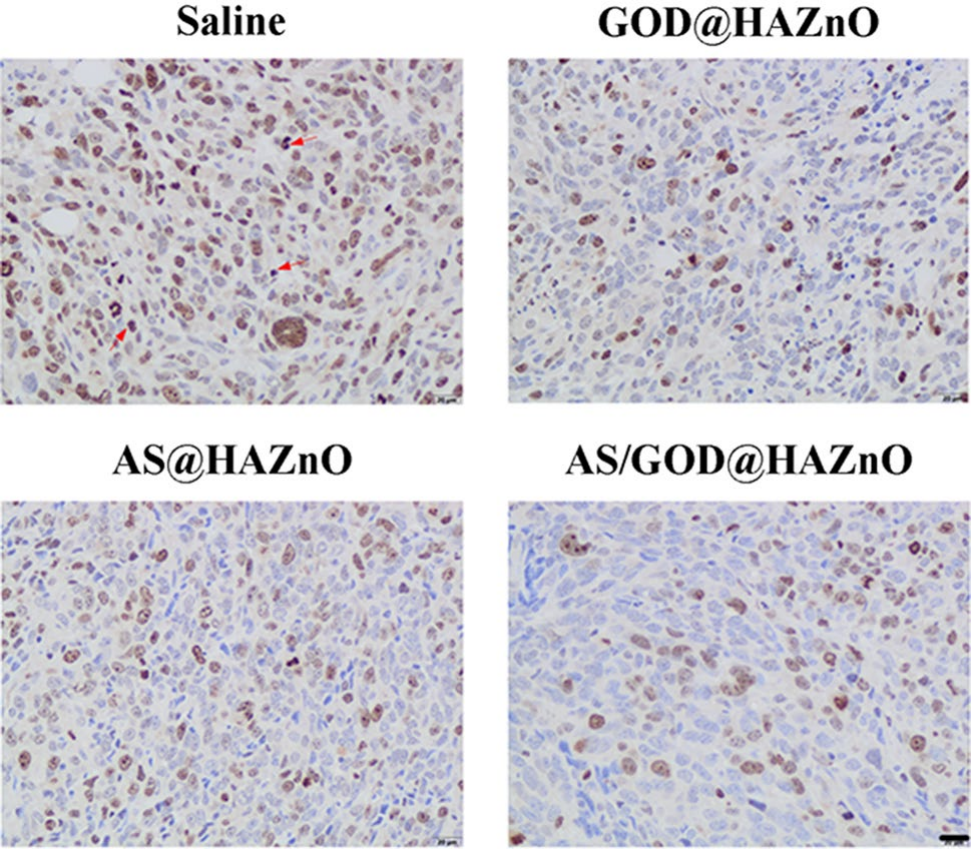
The IHC analysis result of tumor tissues from mice treated with saline, GOD@HAZnO, AS@HAZnO, and AS/GOD@HAZnO NPs. Scale bar = 20 μm.

Ki-67 is a nuclear antigen that is related to cells’ proliferation process and is mainly used for tumor inspection and judging the degree of malignant proliferation of tumors. Simply, the lower the value of Ki-67, the stronger inhibition on tumor growth and the better the chemotherapy effect^27^. As shown in Fig. 10, a large quantity of Ki-67 was expressed in the saline group, indicating that the tumor is in fast proliferation. After treated with GOD@HAZnO and AS@HAZnO, Ki-67 slightly decreased which means the tumor growth encountered inhibition. Besides, Ki-67 in AS/GOD@HAZnO group significantly reduced and cells with a lower proliferation rate took the predominant part. Overall, the combination of starvation therapy and oxidative damage through co-delivering AS and GOD by HAZnO can effectively inhibit tumor growth and send tumor cells to fatal apoptosis.

Cancer starvation therapy is an emerging approach to inhibit tumor growth by blocking nutrient supply. Using GOD to selectively consume glucose in the tumor, thereby cutting off the energy supply of the tumor, can achieve starvation therapy^28^. Compared with before treatment, the ATP concentration in the tumor was significantly decreased after administration (Fig. S3), indicating that AS/GOD@HAZnO played a role in starvation therapy^29^.

### Biosafety evaluation

**Figure 11:**
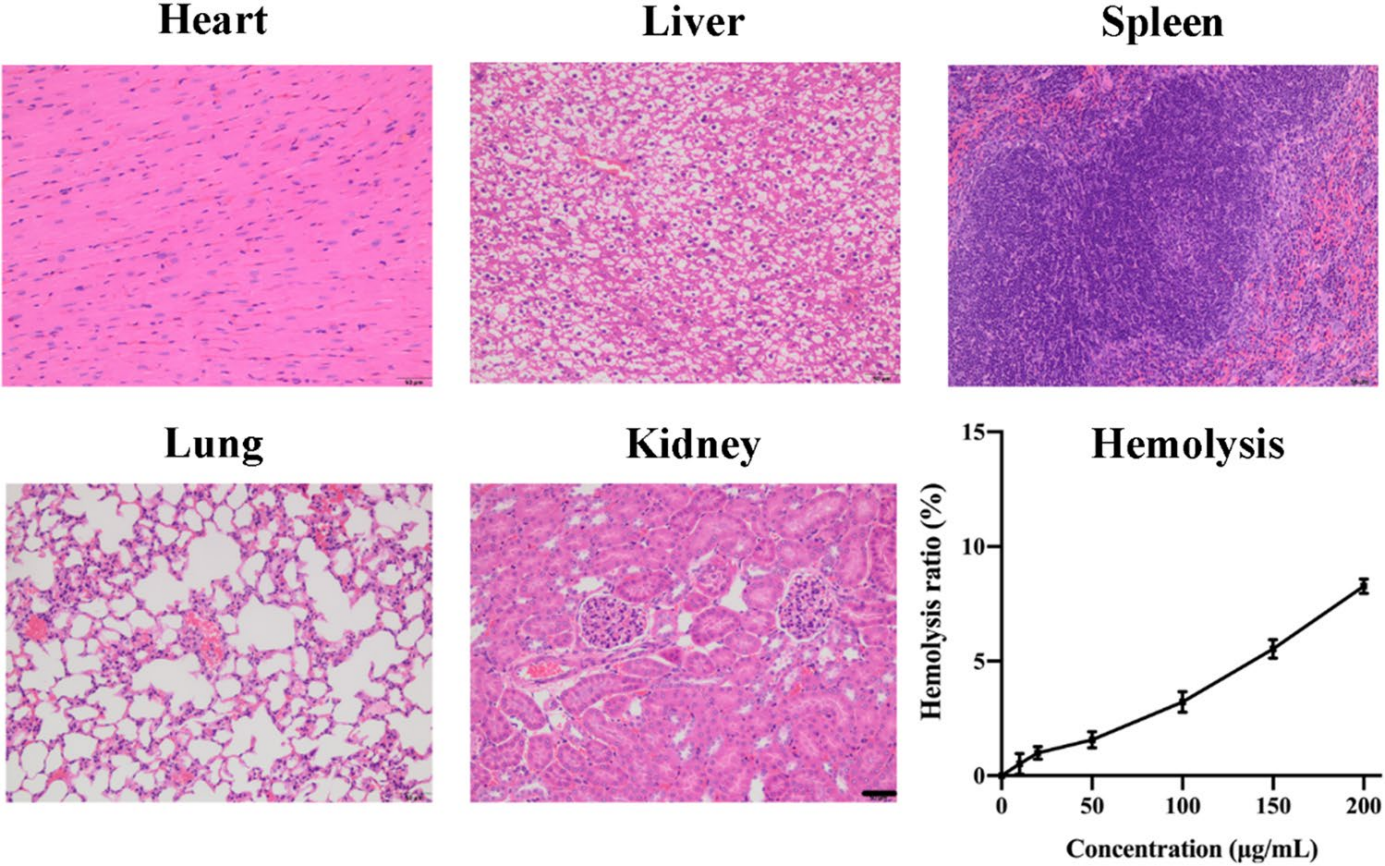
The HE staining result of major organs from mice treated with AS/GOD@HAZnO NPs, Scale bar = 50 μm. The curve represents the hemolysis ratio of AS/GOD@HAZnO NPs at different concentrations.

As illustrated above, the smaller size of ZnO particles would bring stronger cytotoxicity. It is hence highly necessary to evaluate the biosafety of HA-functionalized ZnO NPs. The staining result of major organs was presented in Fig. 11: (1) No cardiac muscle fibers loss was observed in the myocardium and the fibers arranged in normal spiral-like pattern; (2) Hepatocyte nucleus is clear and perfectly round, and the central veins are clear; (3) Abundant splenic corpuscles were observed and there is no morphological changes in the white pulp area; (4) Alveolar sac and alveolus arranged in tight junction; (5) Healthy micro-artery and renal corpuscles were clearly observed. In summary, AS/GOD@HAZnO NPs did not result in apparent pathological changes.

The hemolysis curve also proved that even at a concentration of 200 μg/mL the hemolysis ratio is still under 10%, which suggests that AS/GOD@HAZnO NPs has proper biosafety.

## Conclusion

To sum up, a strategy to combine ROS-based oxidative damage and starvation therapy was developed to realize improved cancer treatment. The functionalization of HA Using HAZnO NPs as delivery vehicles not only realized the acid-responsive release of AS, but also greatly increased the production of ROS and enhanced the AS’s tumor inhibition effect. Meanwhile, the co-loading of GOD helped AS/GOD@HAZnO perform superb glucose catalysis in vitro and efficiently block the nutrient supply of tumor cells. In vivo anti-tumor test attested to the robust tumor inhibition of AS/GOD@HAZnO, and the Tunel and IHC images demonstrated the strong cancer-killing effect of AS/GOD@HAZnO. Such a strategy is proved to be able to make up for the AS’s deficiency in ROS-inducing ability and enhance its anti-cancer effect with multimodal therapies, bringing new possibilities for AS to be clinically applied in cancer therapy.

## Materials and methods

### Materials

Artesunate (AS, 99%) powder was purchased from Shanxi Guanchen Biotechnology Co., Ltd., China); Hyaluronic acid (HA, Mw: 8000 ~ 15,000) was bought from Sigma-Aldrich Chemical Reagent Co., Ltd., USA; Zinc acetate dihydrate (AP, ZnAc_2_·2H_2_O), sodium bicarbonate (AP, NaHCO_3_), and sodium hydroxide (AP, NaOH) were supplied by Sinopharm Chemical Reagent Co., Ltd., China; Glucose oxidase (GOD, 100 U/mg), Adipic dihydrazide (ADH), 1-ethyl-3(3-dimethyllaminopropyl) carbodiimide hydrochloride (EDC·HCl), and 3,5-dinitrosalicylic acid (DNS, 98%) were bought from Aladdin Chemical Reagent Co., Ltd., Shanghai, China. Dialysis bag (MWCO = 3500 Da) was supplied by Bio-Channel Biotechnology Co., Ltd., China.

### Preparation of AS/GOD@ZnO NPs

The ZnO NPs were prepared via the method previously reported with slight modifications^30,31^. First, zinc acetate dihydrate (2 g, ZnAc_2_·2H_2_O) was completely dissolved in 60 °C methanol (20 mL) under mild stirring (water bath). Meanwhile, sodium hydroxide methanol solution was prepared through dissolving 1 g NaOH in 10 mL methanol. The basic solution was added dropwise to the ZnAc_2_ solution under vigorous stirring (60 °C water bath) and the reaction was maintained for 12 h. AS (5% NaHCO_3_ solution, 5 mL) and GOD (aqueous solution, 5 mL) were added, and the mixture was stirred for another 12 h. Then, the mixture was centrifuged at 10,000 rpm for 10 min and the sediment was collected and washed by ethanol for three times. Finally, the nanoparticles were dried in a vacuum oven for 12 h and the AS/GOD@ZnO NPs were obtained.

For the HA functionalization, a facile precipitation method was adopted with slight modifications^32^. HA powder (0.2 g) was fully dissolved in 10 mL deionized water. The previously obtained AS/GOD@ZnO NPs were added to the HA solution under magnetic stirring till fully mixed and then. Next, 15 mg ADH (Adipic dihydrazide) and 7 mg EDC·HCl (1-ethyl-3(3-dimethyllaminopropyl) carbodiimide hydrochloride) were dispersed in adequate water and the ADH/EDC solution was added to the HA/ZnO mixture under magnetic stirring. During stirring, 15 mL methanol was dropped into the reaction system. After 30 min, all the mixture were centrifuged, and the precipitate was collected. After 12 h vacuum drying, the AS/GOD@HAZnO NPs were collected.

### Characterization of AS/GOD@HAZnO NPs

The morphology of AS/GOD@HAZnO NPs was observed by transmission electronic microscope (TEM, HITACHI, Japan). The diameter was measured via dynamic light scattering (DLS, Brookhaven Instruments, Nanobrook 90Plus) in pH 7.4 PBS. FTIR spectroscopy of AS/GOD@HAZnO NPs was carried out by a Micro-Fourier Transform Infrared spectrometer (Nexus 670, USA). The size stability of HAZnO NPs was evaluated by DLS.

### Loading capacity of AS/GOD@HAZnO NPs

Briefly, 50 mg of AS/GOD@HAZnO NPs was dispersed in 20 mL (pH 5.5) PBS solution. The suspension was placed in ultrasonication for 6 h. After centrifugation, the supernatant containing AS and GOD was collected, and the concentration of GOD was evaluated by BCA protein assay. The concentration of AS was measured via the UV–Vis method at 290 nm.

### AS release kinetics

To test the acid-responsive ability of AS/GOD@HAZnO NPs, the AS release behavior was investigated in PBS buffer with pH 7.4, 6.8, and 5.5 respectively through dialysis method. First, 20 mg AS/GOD@HAZnO NPs were transferred to dialysis bags (MWCO = 3500 Da) containing different PBS (50 mL). All dialysis bags were immersed in respective PBS under mild magnetic stirring for 24 h. At each pre-determined timepoints, 2 mL dialysate was taken, filtered with 0.22 μm membranes, and measured at 290 nm by UV–Vis spectrometer. Then 2 mL fresh dialysis medium was replenished into the releasing medium.

### Catalytic activity of GOD@HAZnO NPs

To estimate the starvation therapy efficacy, in vitro GOD (loaded into GOD@HAZnO NPs) catalysis test was performed. First, GOD@HAZnO NPs and free GOD were added into pH 5.5 glucose solutions (glucose concentration = 1 mg/mL, 50 mL) separately. The mixture was immersed in a water bath at 37 °C under magnetic stirring (100 rpm). At pre-determined timepoints, specimens were sampled (2 mL) and same amount of fresh glucose solution was replenished. The glucose level was detected by using the 3,5-dinitrosalicylic acid (DNS) method^33^. The absorbance was measured via UV–Vis method.

### Cell culture

Mouse breast cancer cells (4T1) were incubated in RPMI 1640 medium with 10% FBS and 1% antibiotics (penicillin–streptomycin, 50 U/mL). Cells were cultured in a humidified environment (RH = 90%) with 5% CO_2_ at 37 °C. Cells in their logarithmic phase were adopted for further experiment.

### Cellular uptake and HA competition assay

First, FITC was used as the fluorescence indicator and the FITC-loaded HAZnO NPs were prepared via the method talked above. 4T1 cells were then seeded in a 24-well plate (200 μL cells suspension, 1 × 10^4^ cells per well). Plate was placed in incubator to be incubated for 24 h and upper medium was replaced with fresh medium containing FITC@HAZnO NPs. Another 4 h-long incubation was performed and then cells were washed with icy PBS. The uptake behavior was observed by fluorescence microscope. Blank medium was set as control group.

Receptor blocking analysis studies were performed in selected 4T1 cells that highly express CD44^34^. An excess of HA (1 mg/mL) was added to the cell culture medium for 1 h prior to the addition of FITC@HAZnO and cells co-incubated. The uptake was then observed with a confocal microscope at the indicated times and the fluorescence intensity was quantified with ImageJ software.

### Intracellular ROS assay

ROS is the crucial factor to the oxidative damage from ZnO and AS. A DCFH-DA probe was utilized to indicate the amount of intracellular ROS. Specifically, 4T1 cells were seeded into 6-well plates and incubated for 24 h at a density of 1 × 10^4^ cells per well. Fresh medium with AS/GOD@HAZnO NPs, AS, and HAZnO NPs were then added to replace the previous medium. After another 12 h-long incubation, all cells were rinsed with icy PBS twice and treated with DCFH-DA (10 μM) for 30 min. Cell fluorescence was detected by inverted fluorescence microscope.

Meanwhile, to test the time-dependency of ROS generation, 4T1 cells after preliminary incubation were cultured with AS/GOD@HAZnO NPs following the previous procedures. After 2, 4, and 8 h, cells were rinsed with icy PBS twice and treated with DCFH-DA (10 μM) for 30 min, respectively. Cell fluorescence was detected by inverted fluorescence microscope.

### In vitro cytotoxicity assay

Good biocompatibility is the basis of nanocarriers to be applied in vivo. This section assessed the cytotoxicity of HAZnO NPs by MTT assay method. 4T1 cells were first seeded into a 96-well plate at a density of 1 × 10^4^ cells per well and incubated for 24 h. Previous medium was replaced with fresh RPMI 1640 medium with HAZnO NPs and ZnO NPs respectively. The cell viability was detected via the standard methyl thiazolyl tetrazolium (MTT) assay method after an 24 h incubation.

Besides, 4T1 cells were also used to estimate the inhibition effect of AS/GOD@HAZnO NPs. In detail, 4T1 cells were first seeded in 96-well plates at a density of 1 × 10^4^ cells per well and incubated for 24 h. The culture medium was replaced with fresh RPMI 1640 medium with different concentrations of AS/GOD@HAZnO NPs. Cell viability was tested at 1, 4, 12, and 24 h respectively via the standard methyl thiazolyl tetrazolium (MTT) assay method^35^. Untreated cells were set as control.

The cell viability was calculated by the following formula:

$$ {\text{Cell}}\; {\text{viability}} \left( \% \right) = \frac{{\left( {{\text{OD}}_{{{\text{sample}}}} - {\text{OD}}_{{{\text{blank}}}} } \right)}}{{\left( {{\text{OD}} _{{{\text{control}}}} - {\text{OD}}_{{{\text{blank}}}} } \right)}} \times 100 $$

### Cell apoptosis assay

The apoptosis and necrosis assay of 4T1 cells treated with AS/GOD@HAZnO NPs were carried out by Annexin V-FITC/PI double staining method. 4T1 cells were seeded into 6-well plates at a density of 5 × 10^4^ cells per well and incubated for 24 h. The culture medium was replaced with fresh RPMI 1640 medium with AS/GOD@HAZnO NPs, AS@ZnO NPs, and HAZnO NPs, separately. Untreated cells were set as a control group. After 24 h-long incubation, all cells were stained with 5 µL of Annexin-V-FITC and 10 µL of PI for 30 min. The apoptosis percentage was quantified by flow cytometry.

### In vivo anti-tumor test

Murine breast cancer cells (4T1 cells) and BALB/c mice were here used as the cancer model to establish tumor-bearing mice model. Specifically, 12 female mice (18 ~ 22 g) were fasted for 8 h and randomly divided into 4 groups (n = 3). When the tumor volume grew to 200 mm^3^, GOD@ZnO, AS@ZnO, and AS/GOD@ZnO were injected into mice through tail veins on a AS dosage of 5 mg/kg for consecutive 10 days, respectively. Tumor volume was measured and calculated everyday via the formula: V = (a × b^2^)/2 (a and b are the longest and shortest diameters of the tumor, respectively). Finally, all mice were sacrificed, and tumors were weighed and immobilized for immunohistochemical (IHC) and tunnel test. In addition, tumor tissue of AS/GOD@ZnO was homogenized, lysed, and the supernatant was taken, and then the ATP content in the supernatant was measured using an ATP kit (Beyotime Biotechnology, China) to evaluate the effect of tumor starvation. All animal tests were performed abiding by the principles of the Institutional Animal Care and Use Committee of the Animal Experiment Center of The Third Affiliated Hospital of Jinzhou Medical University (Liao Ning, China).

### Histological analysis

Healthy BALB/c mice (5–6 weeks) were selected and injected with AS/GOD@HAZnO NPs (AS dosage = 3 mg/kg) through the tail veins After 7 consecutive days, all mice were sacrificed, and the major organs (including heart, liver, spleen, lung, and kidney) were collected. Major organs were sliced and sent for histological analysis (H&E staining).

### Hemolysis analysis

Fresh murine blood was first collected from 6 healthy female BALB/c mice and then centrifuged at 1200 rpm for 10 min. The deposited red blood cells (RBC) were washed with normal saline and dispersed into 0.9% saline to form 2% RBC suspension. Next, the red blood cell suspension was treated with AS/GOD@HAZnO NPs of gradient concentrations at 37 °C for 1 h. Then, the samples were centrifuged at 1500 rpm for 10 min, and the supernatant was detected using the UV spectrophotometer for absorbance.

### Statistical analysis

All data were checked by SPSS Statistics 17.0 software and demonstrated as mean ± standard deviation. Student’s t-test was conducted to reveal the differences between two independent groups. Values with *P < 0.05, **P < 0.01, and ***P < 0.001 were, respectively, defined statistically different, significantly different, and very significantly different.

### Ethics approval and consent to participate

All protocols and procedures related to the sampling, care, and management of animals were approved by the Animal Ethics Committee of the Institutional Animal Care and Use Committee of the Animal Experiment Center of The Third Affiliated Hospital of Jinzhou Medical University (Liao Ning, China). All experiments and samplings were carried out in accordance with ethical and biosafety protocols approved by Hospital guidelines. Besides, this study is reported in accordance with ARRIVE guidelines (https://arriveguidelines.org).

## Supplementary Information

Supplementary Information.
