# Supplementary material for: Hyaluronic acid functionalized ZnO nanoparticles co-deliver AS and GOD for synergistic cancer starvation and oxidative damage
Source: Sci Rep. 2022 Mar 17;12:4574. doi: 10.1038/s41598-022-08627-w (PMC8931118; doi:10.1038/s41598-022-08627-w)
Supplement: Supplementary file 1 — Supplementary Information. [file 41598_2022_8627_MOESM1_ESM.docx]

***Supplementary information***

**Hyaluronic acid functionalized ZnO nanoparticles co-deliver AS and GOD for synergistic cancer starvation and oxidative damage**

**Zhenkun Ren^1^, Xibin Han^2^, Lixin Wang^3^, Yi Wang^1*^**

(1. The Third Affiliated Hospital of Jinzhou Medical University, Jin Zhou 121000, P. R. China; 2. The Laboratory Animal Center, Jinzhou Medical University, Jin Zhou 121000, P. R. China; 3. Husbandry and Veterinary Academy, Jinzhou Medical University, Jin Zhou 121000, P. R. China)

* Corresponding author: Email address: Wang Yi ([WY4267583123@163.com](mailto:WY4267583123@163.com))

Tel: +0086-18741653620

**List of figures**


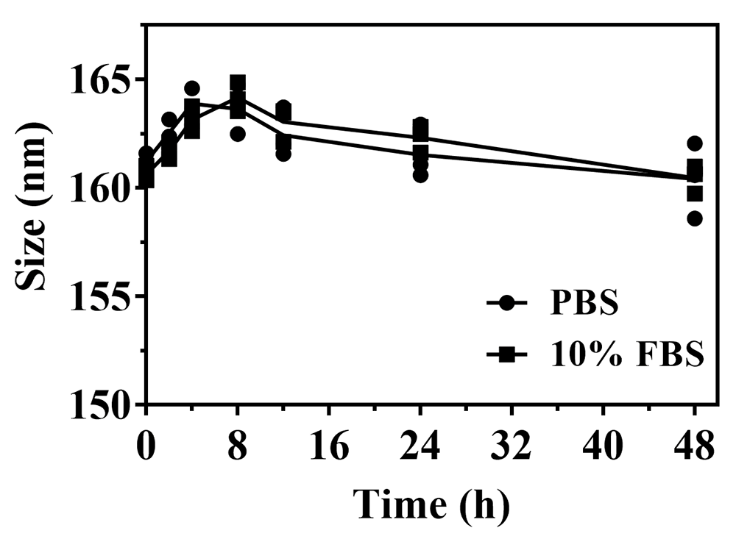


**Figure S1** The size stability of HAZnO NPs in PBS and 10% FBS/PBS for three days at 37 ℃. Data are presented as mean ± SD (n = 3).


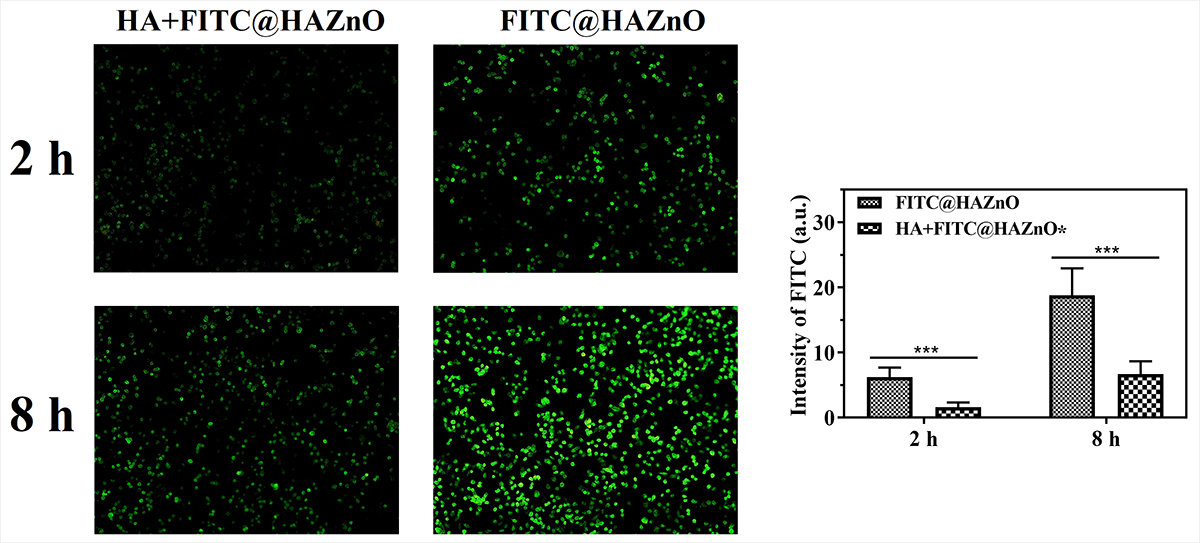


**Figure S2** 4T1 cells after being incubated with FITC@HAZnO and FITC@HAZnO (with HA-pretreated) for different times. Analysis of the FITC fluorescence value by image j. Results are expressed as the mean ± SD (n = 12).


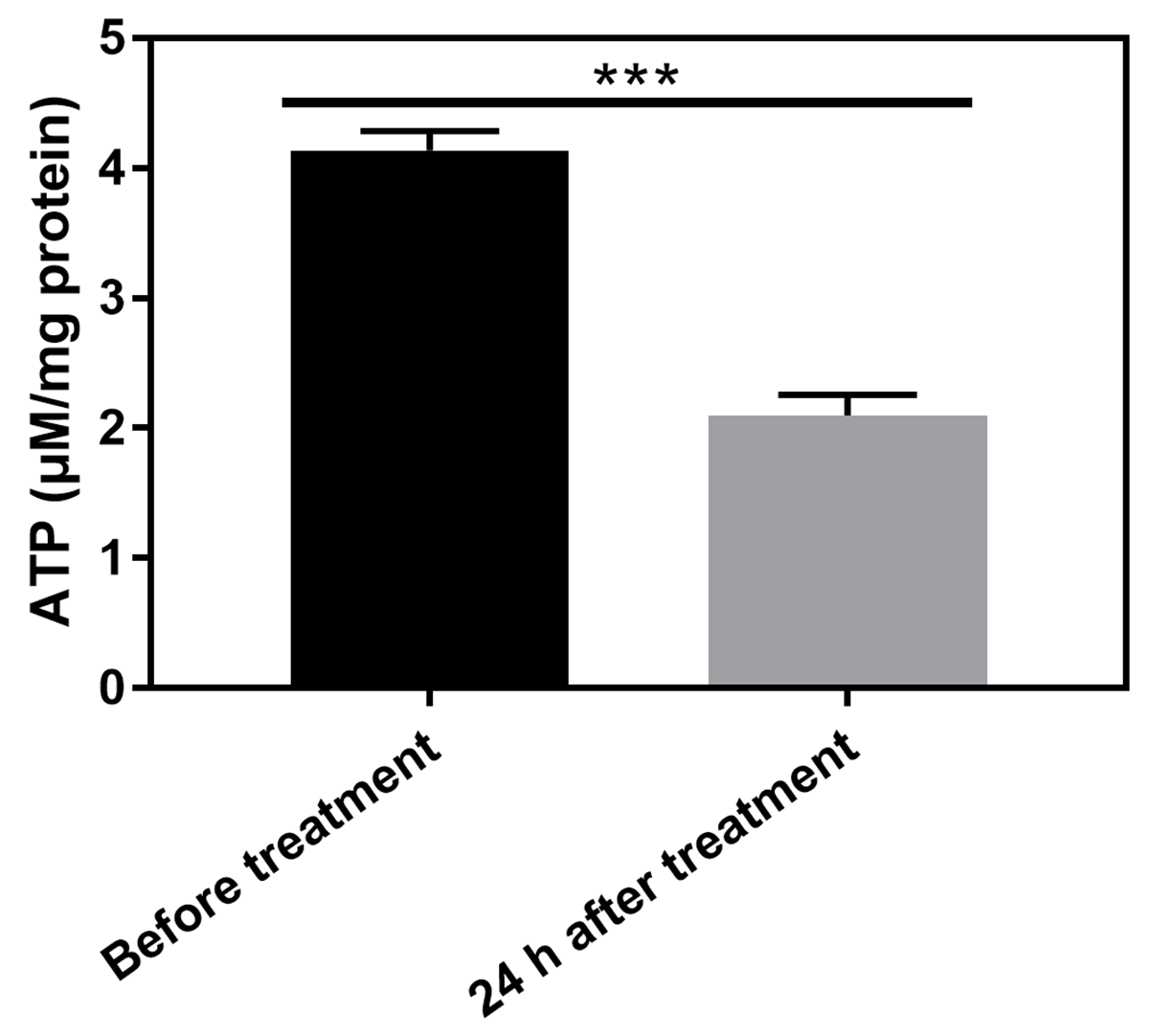


Figure S3 ATP in the tumor tissues from xenograft models treated with AS/GOD@HAZnO (*n* = 3, **P* < 0.01)

**Table S1** Effect of weight ratios of HA to AS/GOD@ZnO on Size and PDI index of AS/GOD@HAZnO. Data are shown as the means ± SD (*n* = 3).

| HA : AS/GOD@ZnO (*w/w*) | Sizes (nm) | PDI |
| --- | --- | --- |
| 8:1 | 269.65 ± 14.17 | 0.12 ± 0.03 |
| 4:1 | 163.33 ± 5.65 | 0.10 ± 0.04 |
| 2:1 | 153.61 ± 4.12 | 0.24 ± 0.07 |
| 1:1 | 147.21 ± 3.04 | 0.32 ± 0.05 |

**Table S2** The size, zeta potential, and PDI value of ZnO、HAZnO and AS/GOD@HAZnO. Data are shown as the means ± SD (*n* = 3).

| Samples | Sizes (nm) | PDI | zeta potential (mV) |
| --- | --- | --- | --- |
| AS/GOD@HAZnO | 163.33 ± 5.65 | 0.10 ± 0.04 | -11.53 ± 1.01 |
| HAZnO | 161.72 ± 4.36 | 0.09 ± 0.03 | -8.40 ± 1.15 |
| ZnO | 144.71 ± 4.80 | 0.27 ± 0.04 | 14.64 ± 0.87 |

**Table S3** Quantitative analysis of the total population of apoptotic cells for each group. Data are shown as the means ± SD (n = 3).

| Samples | Q1 (Necrotic) | Q2 (Late apoptosis) | Q3 (Early apoptosis) | Q4 (Viable cells) |
| --- | --- | --- | --- | --- |
| Control | 0.94 ± 0.03 | 0.60 ± 0.07 | 1.85 ± 0.93 | 96.57 ± 0.90 |
| GOD@HAZnO | 0.35 ± 0.09 | 1.27 ± 0.23 | 16.00 ± 2.15 | 82.37 ± 2.11 |
| AS@HAZnO | 1.03 ± 0.30 | 4.23 ± 0.26 | 24.30 ± 4.73 | 70.20 ± 4.75 |
| AS/GOD@HAZnO | 0.14 ± 0.10 | 1.97 ± 0.65 | 38.80 ± 17.17 | 59.10 ± 17.04 |
